# Air Chambers

**Published:** 1877-11

**Authors:** J. R. Clayton

**Affiliations:** Shelbyville, Ind.


### AIR CHAMBERS.

BY J. R. CLAYTON, D. D. S. SHELBYVILLE, IND.

There is no hing so generally used, and nothing so entirely useless as air chambers, as they are called. They are not only useless, but positively injurious.

I am convinced, that much of the inflamed condition of the mucus membrane of the mouth, of which we have heard so much since the introduction of rubber for plates, is justly chargable to the air chambers, and not to the rubber itself.

We have a congested condition of the capillaries, and small veins to fill up the chamber, which must be filled before the plate will adhere to the mouth, and after it is filled, we have what we ought to have had at once, simply a fit and nothing more.

The patients may complain of a drawing sensation, and we put them away by saying that the parts will accommodate themselves to the plate. This is true, nature is very accommodating, although we may suffer, but it is at the expense of the tonicity of the small vessels. Who has not seen instances where the blood was almost ready to start from the pores of that semi-fungus tissue, formed by the drawing of the chamber. I have had much less trouble in getting plates to fit, since I have abandoned chambers than ever before, and have yet to see a mouth so congested under a plain plate as one with a chamber. To prove that a chamber is not necessary, take a couple of panes of glass, a couple of oil-stones, or anything that has a smooth and flat surface, wet one end of each, put them together, and see the tenacity with which they hold together. A good fitting plate need leave no marks whatever upon the membrane. I know that we will not have that drawing sensation, when the plate is first put in, and which satisfies the patient, because of the idea that the plate is going to "stick," but after the vessels have yielded, the parts "accommodated" themselves to the plates, there will be no difference in the feeling. With good material for impressions, (I always use wax) cast made plain, and if the mouth is very soft on each side, between palatal spine and tuberosity of the maxilla it may be scraped a little there, we can get good fitting plates, please ourselves and patients better, and have less complaint about rubber and congested mouths.

